# Characterization of *gana-1*, a Caenorhabditis elegans gene encoding a single ortholog of vertebrate α-galactosidase and α-N-acetylgalactosaminidase

**DOI:** 10.1186/1471-2121-6-5

**Published:** 2005-01-27

**Authors:** Jana Hujová, Jakub Sikora, Robert Dobrovolný, Helena Poupětová, Jana Ledvinová, Marta Kostrouchová, Martin Hřebíček

**Affiliations:** 1 Institute of Inherited Metabolic Disorders, Charles University, 1^st ^Medical Faculty, Prague, Czech Republic

## Abstract

**Background:**

Human α-galactosidase A (α-GAL) and α-N-acetylgalactosaminidase (α-NAGA) are presumed to share a common ancestor. Deficiencies of these enzymes cause two well-characterized human lysosomal storage disorders (LSD) – Fabry (α-GAL deficiency) and Schindler (α-NAGA deficiency) diseases. *Caenorhabditis elegans *was previously shown to be a relevant model organism for several late endosomal/lysosomal membrane proteins associated with LSDs. The aim of this study was to identify and characterize *C. elegans *orthologs to both human lysosomal luminal proteins α-GAL and α-NAGA.

**Results:**

BlastP searches for orthologs of human α-GAL and α-NAGA revealed a single *C. elegans *gene (R07B7.11) with homology to both human genes (α-galactosidase and α-N-acetylgalactosaminidase) – *gana-1*. We cloned and sequenced the complete *gana-1 *cDNA and elucidated the gene organization.

Phylogenetic analyses and homology modeling of GANA-1 based on the 3D structure of chicken α-NAGA, rice α-GAL and human α-GAL suggest a close evolutionary relationship of GANA-1 to both human α-GAL and α-NAGA.

Both α-GAL and α-NAGA enzymatic activities were detected in *C. elegans *mixed culture homogenates. However, α-GAL activity on an artificial substrate was completely inhibited by the α-NAGA inhibitor, N-acetyl-D-galactosamine.

A GANA-1*::*GFP fusion protein expressed from a transgene, containing the complete *gana-1 *coding region and 3 kb of its hypothetical promoter, was not detectable under the standard laboratory conditions. The GFP signal was observed solely in a vesicular compartment of coelomocytes of the animals treated with Concanamycin A (CON A) or NH_4_Cl, agents that increase the pH of the cellular acidic compartment.

Immunofluorescence detection of the fusion protein using polyclonal anti-GFP antibody showed a broader and coarsely granular cytoplasmic expression pattern in body wall muscle cells, intestinal cells, and a vesicular compartment of coelomocytes.

Inhibition of *gana-1 *by RNA interference resulted in a decrease of both α-GAL and α-NAGA activities measured in mixed stage culture homogenates but did not cause any obvious phenotype.

**Conclusions:**

GANA-1 is a single *C. elegans *ortholog of both human α-GAL and α-NAGA proteins. Phylogenetic, homology modeling, biochemical and GFP expression analyses support the hypothesis that GANA-1 has dual enzymatic activity and is localized in an acidic cellular compartment.

## Background

Humans have two enzymes with α-galactosidase activity and an acidic pH optimum, α-N-acetylgalactosaminidase (α-NAGA) (previously called α-galactosidase B) and α-galactosidase A (α-GAL). Hereditary deficiency of each of the hydrolases causes a distinct lysosomal storage disorder in humans, Schindler and Fabry diseases, respectively [1,2].

Early studies suggested that both human enzymes were glycoforms with similar substrate specifities. Purified enzymes had similar physical properties, including subunit molecular mass (~46 kDa), homodimeric structure, and amino acid sequences. However, additional studies showed kinetic, structural, and immunologic differences proving that α-GAL and α-NAGA were products of two different genes [3,4]. The two genes differed in the number of exons (7 and 9, respectively) and also in the number, placement, and orientation of Alu repeats. Exons 2 – 7 of the α-NAGA gene showed high similarity to the first six exons of α-GAL gene. Because of the remarkable amino acid identity (49%) and similarity (63%) between the two genes and the similar intron placement, Wang [5] and co-workers suggested that a duplication event occurred during the evolution of both enzymes.

Both enzymes belong to the glycoside hydrolase family 27 clan D [6]. Glycoside hydrolase family 27 clan D orthologs have been identified in a broad spectrum of prokaryotes and eukaryotes, including plants. Members of the family have a highly similar active site and share the same reaction mechanism. The structures of chicken α-NAGA, human α-GAL and rice α-GAL have been determined by X-ray crystallography [7-9]. Chicken and human enzymes have a homodimeric quarternary structure whereas rice α-GAL acts as a monomer. The monomer units are composed of two distinct domains. Domain I contains the active site and adopts a (β/α)_8 _barrel structure, a domain fold observed commonly in glycosidases. Domain II has eight antiparallel β strands, packed into two β sheets in a β sandwich fold containing a Greek key motif [8].

The physiological importance of both enzymes is evidenced by the severe presentation of α-NAGA and α-GAL deficiencies in humans [1,2].

Our recent study on degradation of blood group A and B glycolipids in Fabry cells indicated a high residual activity in Fabry cells toward natural substrate glycolipid B-6-2 [10] although α-galactosidase activity was completely absent when measured in vitro by routine procedures using artificial substrates. We proposed that another enzyme, different from α-GAL, contributes in vivo to hydrolysis of α-galactosides. We suggested α-NAGA as the most likely candidate. Human α-NAGA is known to accept α-galactosides albeit with a high K_m _[11,12]. Its activity must be inhibited when measuring α-GAL in tissues with high α-NAGA activity [13].

We investigated the phylogenesis of a single *C. elegans *α-GAL and α-NAGA ortholog (*gana-1*) to both human genes. We present evidence suggesting that this gene has indeed evolved from the α-GAL/α-NAGA ancestral gene before the duplication event which resulted in separate α-NAGA and α-GAL genes in higher metazoans. We further performed structural analysis of the GANA-1 3D model acquired by homology modeling. We determined the spatial and temporal expression of the gene in transgenic worms using a translational reporter and examined the effect of RNA interference (RNAi) as a first step in the possible use of *C. elegans *as a model organism for Schindler and Fabry diseases.

## Results and discussion

### cDNA amplification and sequencing

The complete *C. elegans *genome [14,15] contains only one open reading frame, designated *gana-1 *(R07B7.11) that has sequence similar to human genes encoding α-GAL and α-NAGA. Similar results were obtained from searching the available *C. briggsae *genome sequence [15]. The *gana-1 *gene consists of 5 exons and 4 introns and is annotated as an ortholog of human α-NAGA. Several EST clones for this open reading frame (ORF) have been reported and open-reading-frame sequence tag (OST) is present in the Worfdb database [16]. Available public database data are in agreement with our findings.

**Figure 1:**
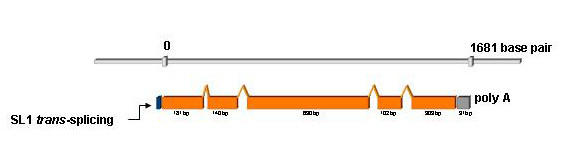
***gana-1 *gene structure. **Schematic representation of *gana-1 *gene structure. The length of genomic DNA from start to stop codons is 1681 bp. The spliced cDNA consists of 1356 bp + 91 bp of 3' UTR.

We verified the gene structure by sequencing the PCR products from genomic DNA and cDNA (Figure 1). The analyzed region spanned the entire coding region and the 3' and 5' untranslated regions (UTR). The 5' UTR SL1 element suggests that the gene is either the only gene transcribed from the promoter or is the first gene in an operon including *gana-1 *and the two predicted downstream genes R07B7.12 and R07B7.13. Although this region has not been reported as an operon, the physical distances between this cluster of three genes are suggestive of an operon [17-19]. No alternative splicing was found using RT/PCR, a feature similar to both human and mouse orthologs. RNA editing was reported in the 3' UTR of human α-GAL, a finding that another group was unable to confirm [20]. We noted no signs of RNA editing in clones derived from the *gana-1 *cDNA.

### Phylogenetic studies

**Figure 2:**
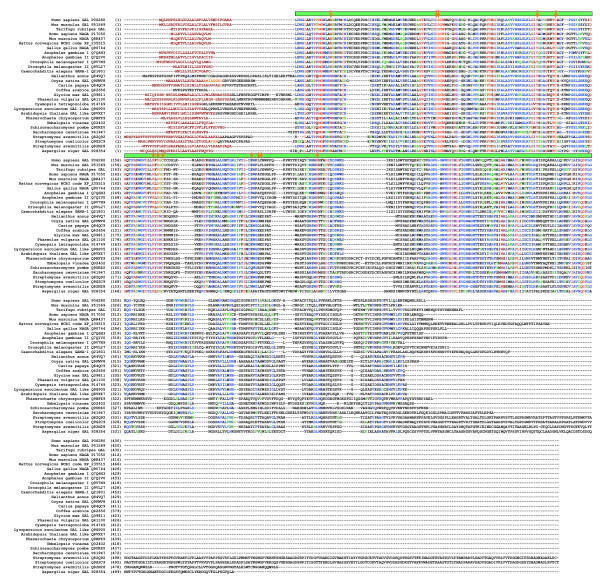
**Multiple alignment. **Multiple alignment of 29 sequences homologous with GANA-1. These sequences represent animal, plant and protozoan kingdoms. The SwissProt/TrEMBL codes are part of the sequence names. Predicted signal peptides are shown in brown letters. In cases where two signal sequence prediction algorithms gave different results the difference is marked by amber color. The residues forming active site pocket of GANA-1 are indicated by arrowheads above the alignment. The catalytic domain I is indicated by green band above the alignment.

**Figure 3:**
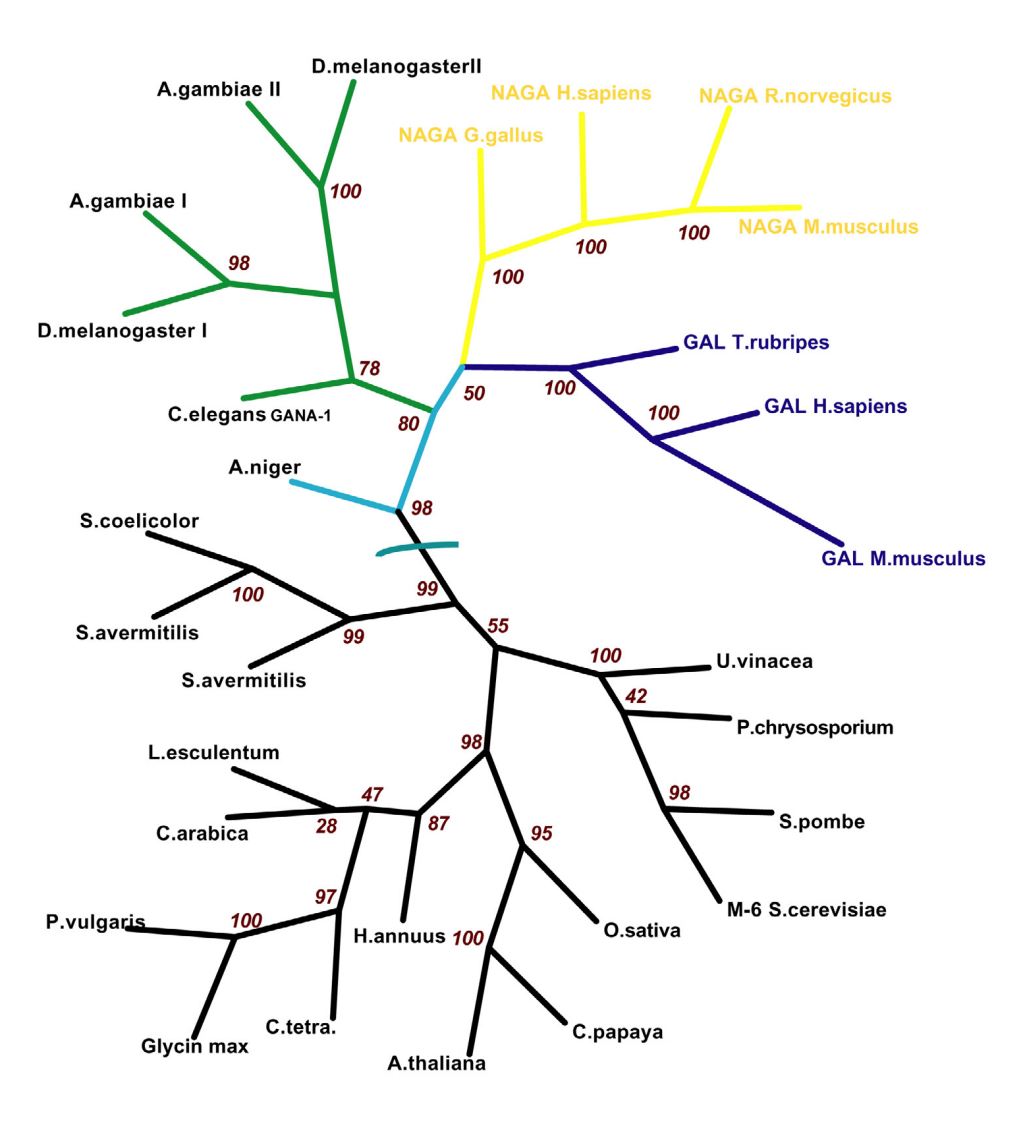
**Cladogram of GANA-1 orthologs. **Cladogram of GANA-1 orthologs. The numbers at the branch nodes represent bootstrap values.

We aligned GANA-1 protein sequence with other melibiase family members (Figure 2) and constructed a phylogenetic tree (Figure 3). The alignment showed a striking similarity of GANA-1 to all other included sequences. GANA-1 had the highest sequence similarity with *Anopheles gambiae *GAL (46%), the lowest similarity was observed with *Streptomyces avertimilis *GAL (22%). The results of our phylogenetic analysis are in accordance with generally accepted evolutionary concepts. The analysis identified four main clades: animal NAGAs, animal GALs, plant/lower organisms GALs and the clade containing sequences of *Drosophila melanogaster*, *Anopheles gambiae *and *Caenorhabditis elegans*. The branch including *C. elegans *is positioned between higher animal GALs and NAGAs and plant/lower organisms GALs. This position in the tree infers the evolutionary ancestrality of *gana-1 *gene to both animal GALs and animal NAGAs. However, this conclusion is not in complete agreement with the presence of pairs of genes in *Drosophila *and *Anopheles *genomes annotated as α-GALs and α-NAGAs. The presence of these genes in the *Caenorhabditis/Drosophila/Anopheles *branch (and not in the GAL and NAGA clades) could be due to low divergence of these sequences from a common ancestral gene or to independent gene duplication in the *Drosophila/Anopheles *ancestral organism. It is also important to note that the phylogenetic analysis by maximum parsimony algorithm placed the *Caenorhabditis/Drosophila/Anopheles *branch into the neighborhood of the animal NAGAs branch [8]. In this case the computational algorithm probably favored the lower number of necessary sequence changes (parsimony) between GANA-1 and NAGA clade sequences.

In our opinion, the phylogenetic analysis provides evidence that the GANA-1 evolved from a common ancestor of α-GAL and α-NAGA enzymes. However, the topology of the tree could also be explained by a loss of the second gene during the evolution of *C. elegans*. In this case the enzyme found in *C. elegans *would probably be the ortholog of human α-NAGA and the lost gene would likely be the ortholog of human α-GAL. The likelihood of these two hypotheses depends on functional divergence of duplicated gene products and their dispensability for organism's metabolic pathways [21].

### Homology modeling

**Figure 4:**
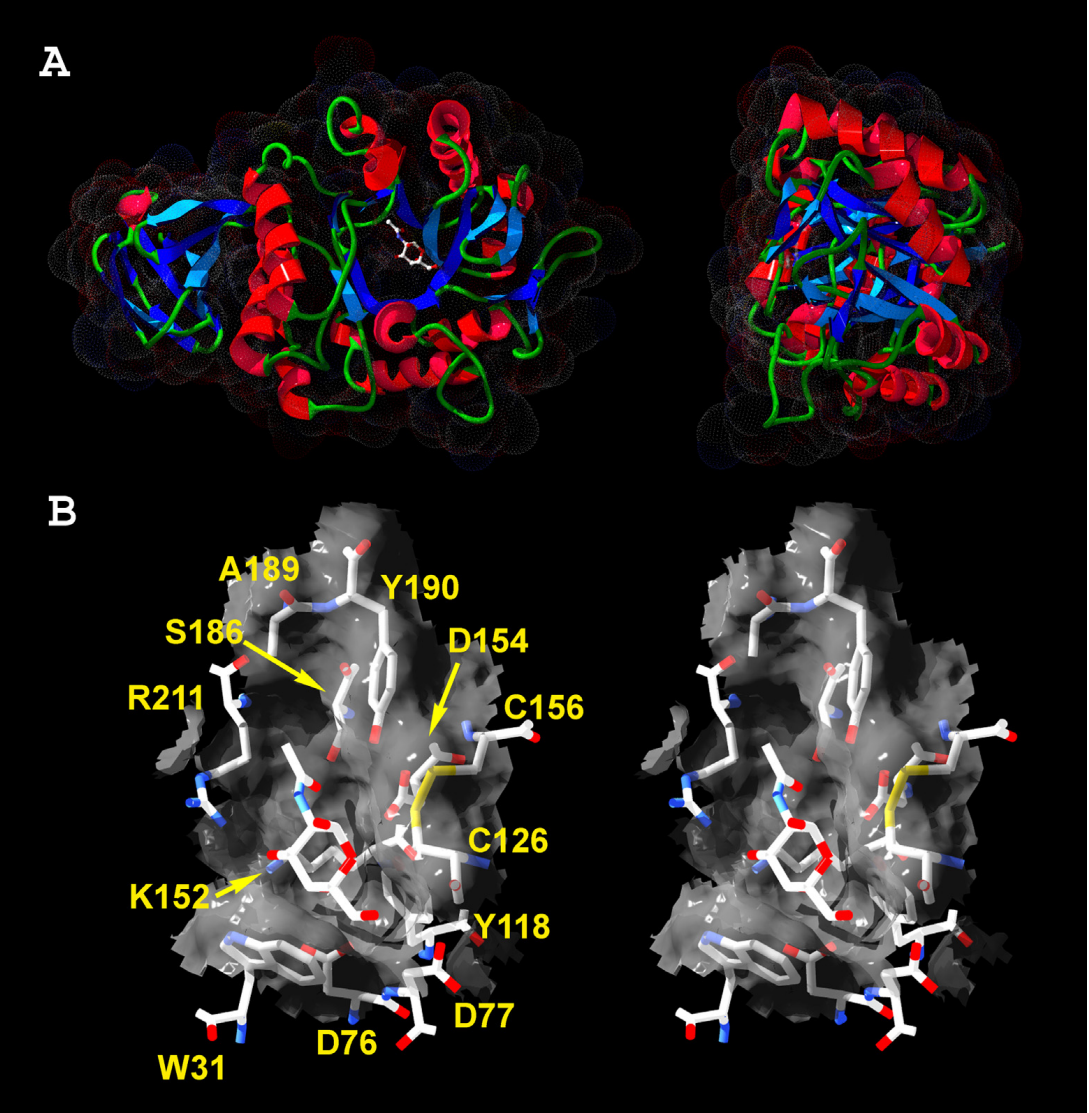
**GANA-1 protein model. **A) Ribbon representation of GANA-1 monomer model. A two-domain structure is apparent in the left picture. The N-acetyl-D-galactosamine (inhibitor) is placed into the active site. Dots represent VdW radii of surface atoms. B) Stereo picture of the active site pocket with N-acetyl-D-galactosamine (inhibitor) and amino acid labels. The viewing angles for stereo representation of the protein structure are ±2 degrees from the central axis.

The best Squared Root of Mean Square Deviations value (RMSD), found between GANA-1 backbone atoms and the chicken α-NAGA template [7], was 0.52 Å. The structural model of the enzyme molecule has a two-domain structure (Figure 4A). Domain I, which contains the predicted active site, adopts a (β/α)_8 _barrel structure which represents a common motif in many glycoside hydrolases. Less conserved is domain II that has a β domain with β sandwich structure containing a Greek key motif. The active site pocket of GANA-1 is formed by the same twelve amino acids (W31, D76, D77, Y118, C126, K152, D154, C156, S186, A189, Y190 and R211) (Figure 4B) as in chicken α-NAGA. This finding infers their identical function in catalytic reactions as described for chicken α-NAGA [7]. D134 carboxyl starts the initial nucleophilic attack and D215 carboxylic oxygen serves as a subsequent donor and acceptor of the proton during the reaction cycle.

Residues forming the "N-acetyl recognition loop" in the chicken α-NAGA [8] (S172, A175, Y176) have the closest contact with the N-acetyl moiety of the ligand. These residues are completely conserved between human and chicken NAGAs, but in human α-GAL serin 172 is replaced by glutamine and alanine 175 is replaced by leucine. The replacements with bulkier residues apparently discriminate between α-GAL and α-NAGA substrates. While NAGAs can accommodate α-galactose and can have some α-GAL activity, GALs do not have α-NAGA activity and are not inhibited by N-acetylgalactosamine. The corresponding residues of GANA-1 in the NAGA recognition loop are S186 and A189 and are characteristic for NAGAs.

According to Garman [8] the key residue in the dimer interface in human α-GAL is F273. Residues corresponding to this position in other orthologs can serve as predictive markers of the protein quartenary structure. Phenylalanine or tyrosine is present in enzymes that act as homodimers while glycin indicates a monomeric structure [8]. The equivalent residue to human α-GAL F273 in GANA-1 is lysine at position 257 which is suggestive of homodimeric structure due to its sterical properties. The homology modeling showed that a groove opposing K257 is formed by residues T260, L261, D262, M263, I389, V390 and V391 of the other monomer unit of GANA-1. In the case of chicken α-NAGA these residues are equivalent to S246, Y247, E248, Q249, N375, P376 and S377 (for details see Additional file 1).

### Biochemical studies

Standard bacteria/nematode separation protocol previously used by other authors [22,23] while evaluating lysosomal enzyme activites is based on sucrose flotation approach. We avoided standard sucrose flotation of worms because we could not exclude unpredictable artifacts caused by this compound, which is known to induce artificial lysosomal storage in eukaryotic cells and to alter lysosomal gene expression at concentrations significantly lower [24] than those used in flotation protocols.

We found both α-GAL and α-NAGA enzymatic activities in the homogenates from *C. elegans *N2 strain using 4-methylumbelliferyl (MU) substrates. The α-NAGA activity was dominant over the α-GAL activity. The activity of α-NAGA measured at 37°C was 430 nmol.mg^-1^.h^-1 ^with MU-α-N-acetylgalactosaminide compared to the activity of α-GAL with MU-α-galactopyranoside of 43 nmol.mg^-1^.h^-1 ^(about 10% of that of α-NAGA).

In the assay of α-GAL, the degradation of the MU-α-galactopyranoside was inhibited up to 95% in the presence of N-acetyl-D-galactosamine (D-GalNAc), whereas in the presence of D-galactose (D-Gal) the degradation of the same substrate was inhibited up to 75%. In the assay of α-NAGA, the degradation of the MU-α-N-acetylgalactosaminide was inhibited up to 97% by D-GalNAc and up to 90% by D-Gal. No inhibition of α-NAGA and α-GAL activity by D-glucose was observed.

According to published observations in human enzymes, D-GalNAc has no inhibitory effect on α-GAL activity. On the other hand, human α-NAGA activity is inhibited by both, D-GalNAc and D-Gal [25]. The model of GANA-1 predicts only one active site per monomer of the enzyme. If the enzyme had activity toward both substrates, MU-α-D-galactopyranoside and MU-α-N-acetylgalactosaminide, it is to be expected that D-GalNAc and D-Gal would inhibit both activities. The strong inhibitory effect of D-GalNAc on the α-GAL activity, which is not present in human α-GAL, supports the hypothesis that *C. elegans *has only one enzyme with both α-GAL and α-NAGA activities. Nevertheless, these experiments were not conducted with the pure enzyme and thus do not provide absolute proof of this hypothesis.

### RNA interference

RNA interference assays directed against the whole coding region of *gana-1*, employing combination of microinjection and feeding approaches, did not reveal any abnormal morphological phenotypes. Nevertheless, measurement of α-GAL and α-NAGA activities in four different experiments showed a simultaneous decrease of both enzymatic activities in RNAi-treated worms (Table 1) as compared with control animals. In all RNAi experiments, both α-GAL and α-NAGA activities decreased proportionally, usually by tens of percent of activity of appropriate controls. The activity of the control enzyme (β-hexosaminidase) did not differ between the RNAi-treated nematodes and controls (data not shown). This finding supports the specificity of *gana-1 *RNAi. The differences between individual experiments are not surprising due to the well-known variability in the efficiency of RNAi [26]. The results of RNAi experiments further support the hypothesis that GANA-1 has both enzymatic activities.

**Table 1:** α-GAL and α-NAGA activities after *gana-1 *RNAi. The table shows a proportional parallel decrease of both enzymatic activities (α-GAL and α-NAGA) after *gana-1 *RNAi compared to controls.

experiment	sample	α-GAL	α-GAL (% of control)	α-NAGA	α-NAGA (% of control)	α-NAGA/α-GAL (% of control)
						
		nmol mg^-1^h^-1^		nmol mg^-1^h^-1^		
1	control	1.78	100	53.13	100	
	*gana-1 *RNAi	1.19	67	26.63	50	0.75
2	control	13.26	100	221.68	100	
	*gana-1 *RNAi	11.1	84	195.13	88	1.05
3	control	3.43	100	61.68	100	
	*gana-1 *RNAi	1.02	30	11.75	19	0.63
4	control	9.6	100	212.1	100	
	*gana-1 *RNAi	2.9	30	50.69	24	0.80

Both enzymatic activities were lower in RNAi-treated and control worms cultured on the bacterial strain HT115 [26] compared to a N2 strain cultured on the OP50 strain.

RNAi previously provided sufficient level of inhibition of structural lysosomal proteins for development of abnormal phenotypes in the worm [27,28]; however, it is apparently not efficient enough for lysosomal catalytic proteins.

### Expression of *gana-1*

To study the expression of *gana-1 *in *C. elegans*, we created transgenic worms with a reporter gene containing the entire coding region of *gana-1 *C-terminally tagged with green fluorescent protein (GFP) under the control of a 3 kb region of the *gana-1 *hypothetical promoter. The presence of the *gana-1::GFP *transgene in the worms was confirmed on the level of genomic DNA, cDNA and protein. However, no GFP signal was observed by fluorescence microscopy under the standard laboratory conditions. As Western blotting showed the presence of fusion protein of the expected size (data not shown), we assumed that the absence of the GFP signal was caused by a pH-dependent quenching of GFP fluorescence, which has neutral to alkaline optimum (pH 5.5–12) [29].

**Figure 5:**
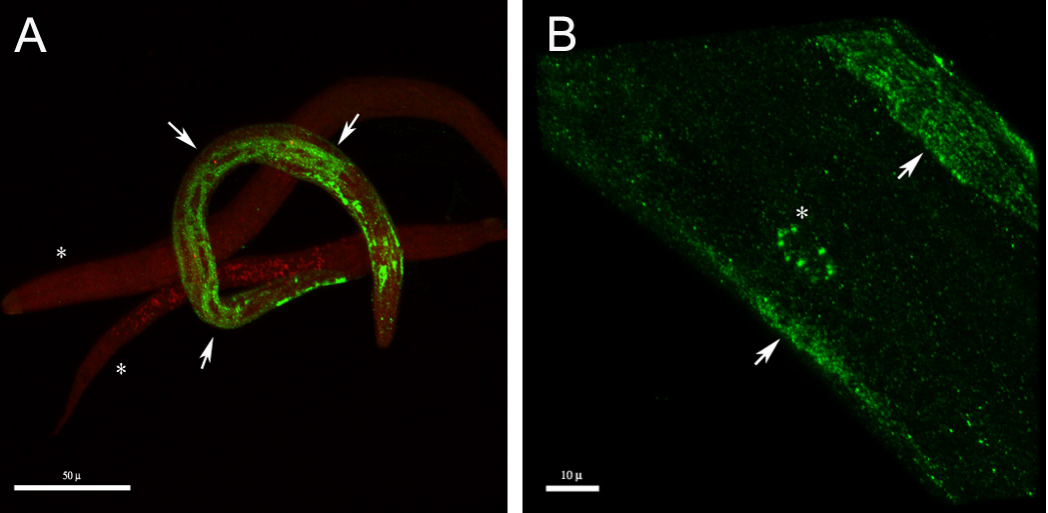
**Immunofluorescence detection of GANA-1::GFP. **A) A coarsely granular cytoplasmic distribution of immunopositivity (green) in body-wall muscle cells (arrowheads). Two non-transgenic worms are shown in the background (asterisks) for comparison. Nuclei are counterstained in red. B) Detailed view of two body wall muscle cells with coarsely granular cytoplasmic distribution of immunopositivity (arrowheads) and a coelomocyte (asterisk), both pictures were acquired by 3D rendering of initial confocal Z-stacks. Note: compare with figure 6.

To study the tissue and intracellular distribution of the fusion protein, we resorted to immunofluorescence detection of the transgene product. Immunofluorescence detection of GFP fusion protein showed a specific and coarsely granular cytoplasmic pattern of fusion protein expression. This transgene product was limited to body wall muscle cells (30% of population) (Figure 5A, Additional file 2) or found in a broader tissue distribution that included body wall muscle cells, intestinal cells and coelomocytes (3–5% of population) (Figure 5B, Additional file 3).

**Figure 6:**
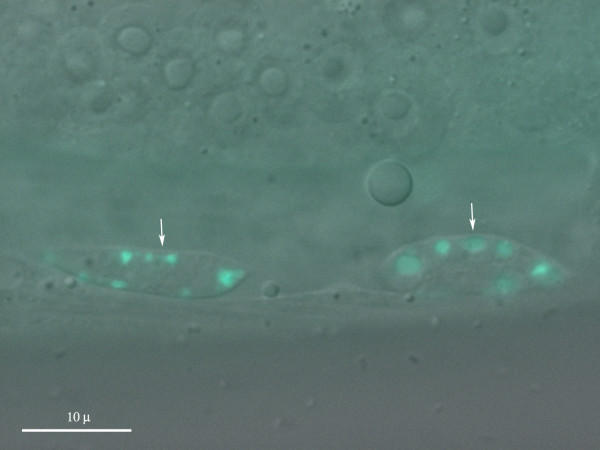
**Alkalization of transgenic worms using CON A. **Two coelomocytes showing a GFP signal in a membrane bound vesicular compartment (arrowheads) after 24 hour incubation in 50 nM CON A. DIC/fluorescence merged image.

This latter staining pattern is consistent with the GFP detection in NH_4_Cl and concanamycin A (CON A) experiments (Figure 6) discussed below. The expression of the transgene was observed in about 30% of the population which corresponded to usual expression efficiency of extrachromosomal array transgenes [30,31]. The immunofluorescence staining protocol resulted in a significant decrease of inherent intestinal granular autofluorescence previously assigned to secondary lysosomes [32]. The decrease of autofluorescence intensity together with its poorly defined emission spectra hampered co-localization study.

To confirm that the absence of the GFP signal was due to the quenching of fluorescence by low pH in the acidic cellular compartment, we used two agents specifically alkalizing acidic cellular compartment [33,34] to enhance the GFP emission. Soaking of *gana-1::GFP *transgenic animals in NH_4_Cl or CON A resulted in a distinct GFP signal in a vesicular compartment of endocytically active coelomocytes in a small proportion of worms (3–5% of population). The GFP signal intensity was dependent on the time of incubation and the concentration of the alkalizing agent used. The first visible GFP signal was observed after 8 hour incubation in 100 mM NH_4_Cl and within 2 hours of incubation in 50 nM CON A. Lower concentrations of both NH_4_Cl and CON A did not result in visible GFP signal even after prolonged incubation of up to 24 hours. The reappearance of the GFP signal after treatment of the worms with compounds increasing the acidic compartment pH indirectly confirms lysosomal localization of the fusion protein. The GFP signal in coelomocytes had the same coarsely granular pattern as that observed after immunostaining.

Limited access of alkalizing agents to the tissues can explain the differences between the results of immunofluorescence and alkalization studies.

## Conclusions

Our findings showed that *gana-1 *is the only ortholog of human α-NAGA and α-GAL in *C. elegans*. Based on phylogenetic and homology modeling analyses we speculate that GANA-1 most probably developed from a hypothetical ancestral gene before the duplication event which gave rise to separate α-NAGA and α-GAL genes.

We also speculate that *gana-1 *gene product has both α-NAGA and α-GAL activities as detected in *C. elegans *homogenates. Importantly, both activities in the worm were inhibited by D-galactose and N-acetyl-D-galactosamine, which is a specific inhibitor of human α-NAGA and does not inhibit α-GAL.

The GANA-1::GFP fusion protein had a pattern of distribution that is compatible with lysosomal subcellular localization. The lysosomal localization of the fusion protein was also supported by pH sensitive fluorescence of GFP that was detectable only after alkalization of the acidic cellular compartment.

Not suprisingly, RNAi of *gana-1 *yielded no abnormal morphological phenotypes, most likely because it did not provide sufficient knockdown of enzymatic activities, necessary for development of lysosomal storage as observed in human pathology states. Nevertheless, *gana-1 *RNAi resulted in a partial decrease of both enzymatic activities supporting the notion that this gene encodes both of them.

It is possible that a deletion allele of *gana-1 *may provide more insight into the function of *gana-1 *and efforts are underway to isolate such alleles. Deletion alleles of lysosomal hydrolases may serve as valuable models of human lysosomal storage disorders.

## Methods

### *C. elegans *methods, strains and nomeclature

The wild type Bristol N2 strain was used for all experiments and was handled under standard laboratory conditions as described previously [35]. Standard methods were used for DNA microinjection [36] and dsRNA synthesis and microinjection [37]. Nomenclature is in agreement with available Genetic Nomenclature for *Caenorhabditis elegans *[15] and has been approved prior to manuscript submission.

### BLAST search

Wormbase (2002–2004 versions and freeze versions [15,38,39]) databases were repeatedly searched for human α-GAL and α-NAGA orthologs using the BLASTP [40] program set at default values. Amino acid sequences of human lysosomal α-GAL and α-NAGA (acc. no. NP_000160 and acc. no. NP_000253 [41]) were used as query sequences.

### cDNA amplification and sequencing

Total RNA was isolated from mixed stages of N2 cultures [42] and reverse transcribed with an oligodT-T7 (5'-AATACGACTCACTATAG) primer and Superscript reverse transcriptase (Invitrogen). The entire coding region of R07B7.11 was PCR amplified in two overlapping PCR products, with intragenic primers designed according to available Wormbase [15] and Worfdb [16] data. SL1 primer (5'GGTTTAATTACCCAAGTTTGAG) and SL2 primer (5'GGTTTTAACCCAGTTACTCAAG) [17] together with gene specific primer (5'ATCCTGATTAATTTTAATTGC) were used to amplify 942 bp of the 5' part of the cDNA and to evaluate the mode of *trans *splicing; the 1142 bp fragment of the 3' end of cDNA was amplified with T7 primer and a gene specific primer (5'CTTAAGTTTGGAATTTATGAA). The dominant PCR products were cloned with TOPO TA cloning kit (Invitrogen) into the pCR 2.1 vector. Positive clones were sequenced using the Li-Cor automated fluorescent sequencer and sequences were aligned with R07B7 reference cosmid sequence in the AlignIR software (Li-Cor) to evaluate splicing boundaries and overall gene organization.

### Multiple alignment and phylogenetic analyses

Confirmed or predicted amino acid sequences of melibiase family members [43] representing plant, unicellular, and animal kingdoms were aligned using ClustalW algorithm [44] and Blosum62 matrix. The SwissProt/TrEMBL [45] accesion code and source organism of the sequences are depicted in Figure 2. The sequence alignment was used for phylogenetic analysis with the software package PHYLIP [46]. The phylogenetic tree is based on 100 bootstraped input alignments and was constructed by maximum likelihood method with Jones-Taylor-Thornton matrix model [47]. Sequence identities between species were calculated without signal sequence in EMBOSS by Needleman-Wunsch global alignment algorithm with Blosum62 matrix, gap penalty – 10 and gap extension penalty – 0.5 [8,48,49]. Signal peptides were predicted at the SignalP server [50] both by algorithms using neural networks and Hidden Markov Models. The results were compared to known signal sequences. The differences between signal peptides predicted by the algorithms are depicted in Figure 2.

The 3D model of GANA-1 is based on the X-ray structure of chicken α-NAGA, rice α-GAL and human α-GAL (PDB codes 1ktcA, 1uas and 1r47, respectively) [7-9,51]. The model was created using the automated homology modeling server SwissModel with structure refinement and model evaluation in the DeepView program [52]. The print quality figures (Figure 4) and animations (Additional file 1) were ray traced using PovRay software package [53].

### Transgenic GFP expression

The entire coding region of the *gana-1 *gene, including 3 kb of its 5'upstream sequence, was amplified from N2 genomic DNA through a nested PCR reaction using DyNAzyme EXT™ PCR system (Finnzymes) and two pairs of primers: the external pair (5'GTGAGAGTGGGGAGATAGAA and 5'TCAATTTGCTTGAGGTACATA) and the internal primers, with overhangs containing SphI and SalI restriction sites respectively (5'ACATGCATGCAACTTTCACAGGAACACAAC and 5'CGACGTCGACAATTGAACTCTATTGGTTCTCAA). The amplified DNA fragment (4709 bp) was cloned using TOPO-XL cloning kit (Invitrogen) into the pCR-XL-TOPO vector. The SphI and SalI *gana-1 *restriction fragment was recloned into the GFP reporter vector pPD95.67 (supplied by A. Fire, Stanford University). The in-frame nature of the insert was confirmed by sequencing. The green fluorescent protein (GFP) fusion construct pJH3 (50 ng/μl) and pRF4 plasmid (50 ng/μl) used as the dominant marker were co-injected into the gonads of young adult N2 worms. Transgenic animals were screened for GFP signal. Nikon Eclipse E800 with C1 confocal module and 488 nm and 543 nm lasers and differential interference contrast (DIC) optics was used for specimen examination. EZ-C1 software (Nikon) was used for picture analysis and 3D rendering (Additional Files 2, 3).

### Alkalization of acidic cell compartment

Mixed stage pJH3 and N2 (control) cultures were harvested from NGM OP50 plates and washed with water. Worms were pelleted by centrifugation (max. 1000 RPM, 2 min.) between the washes. Worms were treated with either one of two agents (NH_4_Cl, concanamycin A – CON A) [33,34], that are known to specifically increase pH in the cellular acidic compartment. For the NH_4_Cl method, animals were suspended in 0, 10, 25, 50, 75 and 100 mM aqueous solutions of NH_4_Cl. Small aliquots of worms were examined after 30 min, 2, 4, 6, 8 and 24 hours. For CON A, animals were suspended in 0, 10, 20, 50, 100, 150, 200 nM solutions of CON A in aqueous media. Small aliquots of worms were examined after 1, 3, 6 and 24 hours.

Microscopical examination was performed as described above.

### Immunofluorescence

The fixation and immunofluorescence staining procedures were based on the approaches of Nonet et al. [54]. Mixed stage N2 cultures were harvested from NGM OP50 plates and washed thoroughly in M9 buffer to remove intestinal bacteria. Worms were pelleted by centrifugation (1000 RPM, 2 min.) between the washes. Worms were fixed overnight in 4% paraformaldehyde in 100 mM sodium/potassium phosphate buffer. Afterwards the pellets were washed three times in 1 × PBS, and incubated in 1% Triton X-100, 100 mM Tris (pH 7.0), 1% β-Mercaptoethanol overnight at 37°C to reduce the cuticle. After 5 washes in 1 × PBS, the worms were incubated for 5 hours in 900 U/ml collagenase type IV (Sigma) diluted in Krebs-Ringer solution (119 mM NaCl, 25 mM NaHCO_3_, 11.1 mM glucose, 1.6 mM CaCl_2_·H_2_O, 4.7 mM KCl, 1.2 mM KH_2_PO_4_, 1.2 mM MgSO_4_·7H_2_O, pH 7.4). The reduction/digestion step was performed twice. Pellets were washed 3 times with 1 × PBS and stored for further processing in AbA buffer (1 × PBS, 0.1% Triton X-100, 1% BSA, 0.05% NaN_3_). AbA buffer was used for antibody dilution. Primary antibody (polyclonal rabbit anti-GFP IgG (Abcam)) was diluted 1:500. Secondary antibody (goat anti-rabbit IgG Alexa Fluor 488 labeled (Molecular Probes)) was diluted 1:1000. Both incubations were performed overnight at room temperature, with AbB buffer (1 × PBS, 0.1% Triton X-100, 0.1% BSA, 0.05% NaN_3_) washes in between.

Nuclei were counterstained with SYTOX orange (Molecular Probes) and the microscopic evaluation was performed as described above.

### Western Blotting

Mixed stage pJH3 and N2 cultures were harvested from NGM OP50 plates. Worms were homogenized by sonication and the concentration of protein was measured by the Hartree method [55]. The proteins (equivalent of 25–50 μg of protein per lane) were separated by SDS-PAGE gradient gel (4% to 20% polyacrylamide) and transferred onto nitrocellulose membrane by semi-dry blotting. The membrane was treated according to a common Western blotting protocol with chemiluminiscence detection (SuperSignal, West Pico) [56]. Rabbit polyclonal anti-GFP IgG (Abcam, dilution 1:5000) was used as the primary antibody, the secondary antibody was goat anti-rabbit IgG/Px (Pierce, diluted 1:20 000).

### RNA mediated interference

The PCR product containing the entire *gana-1 *cDNA was cloned into pCRII-TOPO vector (Invitrogen) and L4440 double promoter vector for microinjection and feeding RNAi respectively. In-vitro transcription employing T7 and SP6 RNA polymerases (Promega) was used to generate antisense single stranded RNA molecules, which were annealed to generate double stranded RNA (dsRNA). dsRNA was microinjected into N2 worms which were fed on HT115 [26]*E. coli *strain carrying L4440 plasmid with *gana-1 *insert. The F_1 _and early F_2 _progeny was screened for morphological phenotypes. N2 worms microinjected with water and fed on HT115 *E. coli *transformed with L4440 vector without insert were used as a control. 5–7 worms were microinjected both with dsRNA and water in each of 4 separate experiments, single worm progeny reaching 110–150 individuals.

### Determination of α-GAL and α-NAGA and β-hexosaminidase activities

Prior to all activity measurements worms were washed from culture plates and repeatedly (6 times) washed and centrifuged in M9 buffer and finally pelleted by centrifugation. 4-methylumbelliferyl (MU)-α-D-N-acetylgalactosaminide (1 mM), 4-MU-α-D-galactopyranoside and 4-MU-β-D-glucopyranoside in theMcIlvaine buffer (0.1 M citrate/0.2 M phosphate buffer at acidic pH) were used as enzyme substrates. Reaction mixtures (sample and enzyme substrate) were incubated at 37°C and reactions were stopped by 600 μl of 0.2 M glycine/ NaOH buffer (pH 10.6) [13,57]. Fluorescence signal of the 4-methylumbelliferone was measured on the luminiscence spectrofotometer LS 50B (Perkin Elmer) (emission 365 nm and excitation 448 nm). Inhibitors (N-acetyl-D-galactosamine, D-galactose and D-glucose) were used in 0.1 M final concentration. All measurements were performed in doublets.

## Authors' contributions

JH carried out molecular, RNAi, expression and biochemical analyses and wrote the first draft of the manuscript. JS participated in the design of all experiments and participated on the bioinformatic, molecular, RNAi and expression analyses and wrote the final version of the manuscript. RD performed phylogenetic analyses including homology modeling. HP participated on the biochemical analyses. MK and JL participated on the coordination of the project. MH conceived the project and provided fundraising. All authors read and approved the final version of the manuscript.

## Supplementary Material

Additional File 1 Structure of GANA-1 dimer. The color of the backbone represents differences of amino acids between GANA-1 and chicken NAGA. Blue color represents identical residues and orange stands for non-conservative changes. The colors from cyan to green represent different degrees of conservation. The surface of one monomer unit at the interface area is rendered with colors representing electrostatic potential. N-acetyl-D-galactosamine (inhibitor) is placed in the active site pocket of both monomer units (D-GalNAc arrowhead). K257 arrowhead depicts predicted dimerisation residue. Click here for file

Additional File 2 Immunofluorescence detection of GANA-1::GFP in muscle cells. 3D volume rendered and animated image corresponding to Figure 5A Click here for file

Additional File 3 Immunofluorescence detection of GANA-1::GFP in muscle cells and coelomocytes. 3D volume rendered and animated image corresponding to Figure 5B Click here for file
